# A multi-faceted approach to sex and gender equity in solid organ transplantation: The Women in Transplantation Initiative of The Transplantation Society

**DOI:** 10.3389/fimmu.2022.1006855

**Published:** 2022-09-26

**Authors:** Roslyn B. Mannon, Elaine F. Reed, Anette Melk, Amanda Vinson, Germaine Wong, Curie Ahn, Bianca Davidson, Bethany Foster, Lori J. West, Katie Tait, Anita S. Chong

**Affiliations:** ^1^ Division of Nephrology, Department of Medicine, University of Nebraska Medical Center, Omaha, NE, United States; ^2^ Department of Pathology and Laboratory Medicine, David Geffen School of Medicine, University of California, Los Angeles, Los Angeles, CA, United States; ^3^ Children’s Hospital, Hannover Medical School, Hannover, Germany; ^4^ Division of Nephrology, Department of Medicine, Dalhousie University, Halifax, NS, Canada; ^5^ Centre for Transplant and Renal Research, Westmead Institute for Medical Research, University of Sydney, Sydney, NSW, Australia; ^6^ Division of Nephrology, National Medical Center, Seoul, South Korea; ^7^ Division of Nephrology, Groote Schuur Hospital, Capetown, South Africa; ^8^ Department of Pediatrics, McGill University, Montreal Children’s Hospital, Montreal, QC, Canada; ^9^ Department of Pediatrics, Alberta Transplant Institute and Canadian Donation and Transplantation Research Program, University of Alberta, Edmonton, AB, Canada; ^10^ The Transplantation Society, Montreal, QC, Canada; ^11^ Department of Surgery, University of Chicago, Chicago, IL, United States

**Keywords:** transplantation, sex, gender, career, outcomes

## Abstract

The advancement of women’s careers in transplantation continues to be challenging. Academic careers in both basic and clinical disciplines in transplantation, such as surgery and management of end organ failure in medical specialties, have been underrepresented by diverse genders and ethnicities. Over the last decade, the Women in Transplantation Initiative (WIT) has solidified to becoming an internationally recognized organization with activities focused on diversity and inclusion in terms of the sexes. The WIT organization is divided into 3 pillars that address career advancement and networking (Pillar 1), scientific investigation and presentations on sex and gender in transplantation (Pillar 2) and investigating and facilitating equitable access to transplantation for women throughout the world (Pillar 3). By taking this multipronged approach of collaborating across continents, leveraging virtual platforms for information dissemination and discussion, and providing financial support for research, WIT has become a highly visible grass roots organization that aims to improve the experience of women as transplant professionals as well as transplant donors and recipients.

## Introduction

In 2005, Professor Kathryn Wood, esteemed immunologist at Oxford University, was elected the first woman President of The Transplantation Society (TTS) in the international Society’s nearly 40-year history. As part of her Presidential mission, Prof. Wood took immediate efforts to change the climate of TTS. While women comprised a sizable component of membership, they were less involved in leadership of committees, and council activities. Her charge was to address specifically the disparity in the participation of women in TTS leadership, as well as career advancement of women in the field of transplantation. By 2009, this goal evolved into a Steering Committee for Women in Transplantation (WIT) to sustainably develop professional opportunities for women transplant professionals. These included “networking” events at major international transplant meetings, facilitating an educational component in those events, and allowing for relationship development amongst women colleagues. At a breakfast networking event in February 2010 at the TTS-sponsored Transplant Transcriptome Meeting in San Francisco, CA, Prof. Wood invited women scientists and physicians attending the meeting to develop a collaborative network to support one another. The intended goal was that women recognize each other’s value by direct advocacy including nominating women as speakers for national and international meetings, and as candidates for recognition awards and leadership positions in national and international societies. With more balanced representation at these prominent positions, it followed that more women would become involved, not only in the professional societies, but in the transplant profession overall. Indeed, an international mentoring program was established in 2011, but barriers to its success included the complex nature of academic centers that differed substantially across different countries, limited abilities to navigate across time zones, and a lack of time of women leaders to take on these activities in addition to their “day jobs”.

In December 2012, after repeated discussion, Prof. Wood proposed requesting primary administrative support from the TTS for WIT to support networking activities, given that women had been, and will continue to be, integral to TTS. Other Steering Committee members, including Drs. Megan Sykes and Nancy Ascher, supported this request, and the WIT initiative under TTS was born. By having current WIT leadership as TTS council members, communication, and continued engagement of WIT in all aspects of the TTS mission are facilitated, particularly in countries where women have limited influence in advancing their careers and limited opportunities, if any, for leadership. Dr. Nancy Ascher became the first WIT chair, and Prof. Wood, co-chair.

At a critical juncture of WIT in 2016, Dr. Elaine Reed, an internationally recognized Immunogenetics and Histocompatibility lab director and human immunologist, was named the Chair of WIT. With financial support from TTS and Novartis, a strategic planning session was convened in May 2017, in Montreal, Canada, inviting members of the WIT Steering Committee. While WIT had been active for 8 years, there was a clear need to accelerate its efforts. Through surveying stakeholders, informational interviews and facilitated activities, a team of women transplant physicians, surgeons, allied health professionals, and scientists expounded and confirmed formally the mission of WIT and developed a structure that articulated formal goals and activities. This vision was comprehensive and critical as the WIT Initiative was becoming more popular and expanding its influence, with more formalized structure and expectations. In this regard, under the leadership of WIT Chair, Dr. Lori West (2018-2020), the mission statement became, “*to advance and inspire women transplant professionals and champion issues of sex and gender in transplantation*” with a vision of world-wide gender equity and inclusiveness in transplantation (https://www.tts-wit.org/).

In the spring of 2021, another review of the strategic plan was led virtually by WIT Chair Dr. Roslyn Mannon (2020-present). The goals were to assess progress and consider modifications. In the course of the review, it was noted that activities related to equitable access to transplantation and the known disparities of females being more likely than males to be living organ donors needed more attention to effect change. This resulted in revision of the pillar structure noted below.

## Pillars and goals

At the conclusion of the May 2017 Montreal meeting, guiding principals were developed based on input of participants to operationalize the initiative. Specifically, WIT would operate as an independent initiative for collaboration and partnership across professional societies and associations who share our mission and goals. WIT would be committed to achieving global diversity in reach, access, and participation to support the WIT mission. As noted above, advocacy, education, engagement and connection across genders, cultures, and professional domains in the field of transplantation were considered critical to the success and impact of WIT.

WIT is unique in that its operational structure is based on three key themes or pillars, and they are shown in **Table 1**.

Pillar I: Advancing and Inspiring Women Transplant Professionals
Pillar 2: Championing Issues of Sex & Gender in Transplantation
Pillar 3: Advocacy and Disparity of Sex in Transplantation.

**Table 1:** Pillars and goals of women in transplantation.

Pillar I	Pillar 2	Pillar 3
Advancing and Inspiring Women Transplant Professionals	Championing Issues of Sex & Gender in Transplantation	Advocacy and Disparity
Enhance women’s participation in the profession: education, networking, and supporting each other (formal and informal mentorship)	Establish sustainable funding for projects (data collection, research, education, etc.) to address issues of sex and gender.	Increase the percentage of female organ transplant recipients (especially in countries with disparities)
Ensure equity in transplantation professional society leadership	Establish educational contributions with designated sessions on issues of sex and gender at major national and international transplant meetings	Decrease disparities in the number of male and female living donors worldwide
Increase the percentage of women speakers in international and national meetings	Extend educational content through virtual mediums	
Support and advocate for monetary grants/award to women transplant professionals.	Lead efforts to publication standards on sex and gender	
**Chairs and Pillar Members (name, origin)**		
**Chair: Anita Chong, USA** **Members** Curie Ahn, South Korea Samantha Anthony, Canada Yolanda Becker, United States Amishi Desai, United States Christine Falk, Germany Hannah Maple, United Kingdom Anjana Pillai, United States Helen Pilmore, New Zealand Vasanthi Ramesh, India	**Chair: Germaine Wong, Australia** **Members** Carla Brady, United States Karen Dwyer, Australia Bethany Foster, Canada Louise Lerminiaux, United States Anette Melk, Germany Rachel Patzer, United States Ruth Sapir-Pichhadze, Canada Maria Simonenko, Russia	**Chair: Bianca Davidson, South Africa** **Members:** Victorine Bandolo, Cameroon Maisarah Jalalonmuhaili, Malaysia Maleeka Ladhani, Australia Louise Lerminiaux, United States Ruth Sapir-Pichhadze, Canada Nicole Scholes-Robertson, Australia Khalida Soki, Kenya Ifeoma Ulasi, Nigeria Amanda Vinson, Canada

Ambitious goals for each pillar were determined by consensus and rounds of voting, as shown in **Table 1**. The pillars were to be sustained by ongoing volunteer efforts in the areas of professional development (Pillar I) and academics contributions (research and training) related to sex and gender (Pillar 2). Indeed, WIT members have developed projects and presentations into enduring materials and highly referenced publications through networking activities or *via* collaboration from developed scientific presentations (1–3). Moreover, Pillar 2 has oversight of a newly developed grant program for trainees and fellows researching the impact of sex and gender in transplantation. With support from industry partners, the intention is to expand this program to support faculty and trainees, so they may develop the necessary expertise and preliminary data for larger grant submissions. Pillar 3, as the newest pillar, has a focus on advocacy for women in low- and middle-income countries. As a provisional project, Pillar 3 members are interviewing women who are transplant recipients, as well as potential candidates for transplantation, to define their journey to transplantation, and identify issues, both medical and psychological, that have impacted this journey.

## Membership

WIT has a membership of 400+ individuals, with the majority of members in North America (42%), followed by Europe (21%), Asia (9%), Middle East and Africa (6%), Latin America (4%) and Oceania (5%); 20% are from low- and middle-income countries. Members are encouraged to join TTS, but this is not required. There is no membership committee and there are no dues. We ask members to provide a brief outline of their expertise with their curriculum vitae and how they wish to contribute to WIT, be it scientific expertise, mentorship, or networking, as part of the WIT mission. Membership is not restricted to physicians and scientists, nor by sex or gender of the participant. New ideas for programs and activities are welcome as well as suggestions for specific speakers outside of transplantation. This format for participation has strengths in that a financial barrier such as dues payments, or requirement for letters of nomination which may be difficult to obtain in male dominated academic organizations. We do encourage male members to join as currently 99% of members identify as women.

## Networking and making connections

Networking events continue to be the foundations of WIT activities and since 2014, there have been 42 events held across the world. Collaboration has always reached beyond kidney transplantation, and a list of the participating organizations and societies is shown in **Table 2**. While more recently pivoting to virtual participation due to COVID-19 suspending in-person meetings, the identification of timely topics of professionalism, sex and gender research, and academic development, have advanced the mission and recognition of WIT. While many have bemoaned virtual platforms, participants have clearly identified that new connections have been created, and the virtual platforms provide regular and more frequent participation than would otherwise not be possible due to limited travel budgets and the reality of frequent travel interfering with professional activities. Indeed, attendance has grown from a total of 455 registrants for 7 in-person events in 2019 to 874 registrants in 16 virtual events in 2021.

**Table 2:** Collaborative organizations with Women in Transplantation.

American Association for the Study of Liver Disease (AASLD)
American Society of Histocompatibility and Immunogenetics (ASHI)
American Society of Nephrology (ASN)/Women in Nephrology
American Society of Transplantation (AST)
Asian Society of Transplantation (AST)
Associação Brasileira de Transplante de Órgãos (ABTO)
Banff Foundation for Allograft Pathology
Canadian Donation and Transplantation Research Program (CDTRP)
Canadian Society of Transplantation (CST)
European Society of Transplantation (ESOT)
Federation of Clinical Immunology Societies (FOCIS)
International Society of Heart and Lung Transplantation (ISHLT)
International Society of Nephrology (ISN)
International Liver Transplantation Society (ILTS)
International Society of Organ Donation and Procurement (ISODP)
International Transplant Science Meeting
International Transplant Nurses Society (ITNS)
International Xenotransplant Association (IXA)
Intestinal Rehabilitation and Transplant Association (IRTA)
Middle Eastern Society of Organ Transplantation (MESOT)
Sociedad de Trasplante de América Latina y el Caribe (STALyC)
The Transplantation Society of Australia and New Zealand (TSANZ)
The Transplantation Society (TTS)

These sessions and meetings developed organically with content and action strategies aimed at engaging the entire transplant community. Examples include an event examining the impact of living kidney donation in women held in partnership with TTS Education Committee in November 2021. This live webinar was developed to examine novel aspects of living donation and included international experts in living donation as well as donors themselves, all of whom identified as women. The timely topics included the psychological and unexpected benefits of living donation and living through the process, highlighting the clinical impact of donation on longer term health and fertility, as well as examining access to live donation and social pressures to do so (4). Another remarkable event held in September 2020 focused on the impact of sex and gender during the COVID 19 pandemic. The online virtual interactions triggered discussion of disparities in transplantation during the COVID-19 pandemic and the impact on careers of woman transplant professionals resulting in a policy viewpoint in *Kidney International* coauthored by session participants (3). In an upcoming interactive symposium on sex- and gender-specific issues in transplantation, we will combine the advantages of both in-person and virtual attendance to explore the needs of future research by taking the female perspective [https://diversity-in-transplantation.org/]. Such meetings support international collaboration and provide opportunities to less experienced women academics to advance their careers, through both formal and less formal mentorship.

## Scholarship

It was recognized by the WIT leadership that making change requires data and developing a body of science to advance. Since its establishment, led primarily by Pillar 2, WIT has developed scientific expertise in sex and gender in solid organ transplantation and transplant outcomes as well as the impact of sex on academic careers. An initial scoping review was developed by colleagues in Pillar 2 (1), including detailed methodology on executing such a review (5). Importantly, WIT members were key coauthors of a detailed review of the impact of sex on all solid organ transplants, calling into action the need for equity for females in the transplant process as well as encouraging investigations into the differences in immunological responses based on sex (6). Inspired by examples such as the Sex and Gender Equity in Research (SAGER) guidelines for publishing (7), Pillar 2 has recently adapted similar recommendations for transplantation that have been endorsed by the journal, *Transplantation* (8).

Recent collaborations with the Asian Society of Transplantation resulted in two highly attended recorded webinars in January and February 2021. The goals of these meetings were to define and characterize the gender inequality issues in the Asia Pacific regions, and to devise strategies to mitigate them. The highlights of this meeting were published (9), which have led to subsequent discussions and collaborations with the Ethics Committee and the Declaration of Istanbul Custodial Group to better document the geographic disparity in transplant access and live donation by women throughout Asia. Additionally, under the leadership of the Asian Society of Transplantation, a voluntary registry has been created with submission of transplant data from 6 counties: South Korea, India, Australia and New Zealand, Philippines, Taiwan, and Japan. The goal of this work is to elucidate the reasons for health disparities, and to define solutions and strategies to reduce and eliminate these by assessing gender differences in living and deceased kidney donation and transplantation across these countries in the Asia-Pacific Region (10). These data demonstrate variations in the frequency of women as living donors and as transplant recipients, dependent on several factors that include the economic status of a particular country, the prevalence of disease in that country, and provision of medical access in that country. While such voluntary registries have biases, it is an important starting point for further data gathering, analysis, and ultimately, policy implementation.

## Challenges to the careers of women in science and transplantation

The challenges for women in science are plentiful, not only in the academic arena but in their considerable social activities and traditional roles as partners and parents, as noted by the recent report of the American Association of Medical Colleges report on Women in Academic Medicine (11). These challenges have been exacerbated by the COVID-19 pandemic (3), resulting in high rates of burn out and withdrawal from the transplant field as in other medical and scientific arenas. Direct efforts to mitigate these challenges were the focus of work by women colleagues in the Transplant Society of Australia and New Zealand (TSANZ), with noted action items for government and societal consideration (12). Pillar 1 has focused on these concerns, with activities to mentor, encourage, and support women in the field of transplantation at all stages. A specific example is the targeted nominations of women for professional society awards that recognize career and scientific excellence. Another intentional Pillar 1 effort is to provide expertise in conference planning to support qualified women speakers and moderators and to prevent “manels”, the all-male scientific panel prevalent at national and international meetings (13). The occurrence of such activities has been so frequent that the National Institutes of Health Director, Dr. Francis Collins, publicly vowed to no longer participate in speaking engagements in which participation lacked gender, ethnic, and racial diversity (14). Another notable new activity developed by Pillar 1 was utilizing the documentary “Picture a Scientist” as a starting point for discussion for both small and large groups (15). This activity resonated across transplant communities globally. The poignancy of the situations of women scientists highlighted across decades of research careers resonated strongly in multiple communities and collaborators (**Table 2**). Support from WIT for a viewing license to provide access to its members and other collaborating organizations has led to group viewings and discussion panels in multiple locations in North America, Europe, Africa, and Asia. The opportunity to engage male colleagues was another important step forward in eradicating disparities in scientific careers in transplantation, that have remained male-dominated for decades.

## Recognizing significant achievements: The women in transplantation awards

Recognition of one’s value and efforts in one’s field is an important aspect of career progression. Frequently, women may be overlooked for these recognitions (16, 17). WIT continues to address this disparity directly with the creation of two achievement awards: (1) the Woman Leader in Transplantation Award, to recognize women who have helped further the field of transplantation through research, policies, leadership, initiatives, or other highly regarded contributions; (2) the Unsung Hero Award presented to women who have had extraordinary impacts in transplantation through community service, volunteering, mentorship, or other community-based activity. Both awards are presented during the Presidential Address at the TTS International Congress biennially. The award winners are highlighted on the WIT website, and a cash award is provided to offset travel expenses to the Congress to receive the award in person. Since 2014, there have been seven women leaders and ten unsung heroes. Likewise, as noted above, Pillar 1 works proactively to identify potential women as candidates of awards from other professional societies, by soliciting and providing nomination packages and letters of support. The impact of these activities has not been included as a metric but will be clearly important moving forward.

## Training the next generation: The WIT grants program

An enormous step forward and an innovative programmatic effort for WIT has been the development of research grant awards for trainees to carry out sex and gender studies in transplantation. The WIT Grants Program, initiated and driven through the tireless efforts of Dr. Roslyn Mannon, was in response to the need recognized by WIT to address key knowledge gaps in the field of sex and gender in transplantation. Not only is there a loss of advancement of women trainees through the career cycle, but additionally, women who stay in academia are less likely than their male counterparts to be lead or senior authors in publications in the field of transplantation (18). While the WIT grants are not limited to female applicants, the aims are to develop new expertise for the applicant, which will be of significant value in promoting more diversity and equity in transplantation. Applicants are required to be in training programs and have identified a mentor who does not need to identify as female but can ensure productivity and sufficient resources to complete the projects. Indeed, current mentors are predominantly but not exclusively female. Essential to the success of this program has been the unrestricted support from enlightened industry partners. Grant competitions have funded four trainees since 2021, with the anticipation of another funding cycle in 2023. Most recently, in collaboration with the Asian Society of Transplantation, WIT is co-sponsoring a competitive award to support data submission and analysis to the Asian Society of Transplantation-WIT registry.

## Discussion

WIT was developed by a group of pioneering women leaders in The Transplantation Society and predates the recent focus on diversity and inclusion across the globe. The successes to date are attributed to the passion and bandwidth to volunteer to support the mission of WIT. The initial goal of WIT was to develop more opportunities for career development *via* networking, collaboration, and mentoring support. The number of collaborations between WIT and with transplant-oriented and organ-specific organizations continue to grow, indicating that needs continue to be unmet. Additional key accomplishments have been the specific focus on gender and sex issues in clinical care and access to solid organ transplantation. This has resulted in impactful published work, and initiation of new registry to address inequalities in Asia. There remains ongoing opportunities and future directions to improve access to transplant care for women (and by women) by informing the public and agencies about the disparate level of female organ donors and recipients, and to continue to support the academic careers of women in transplantation. With an emphasis on advocacy, informal and formal mentorship, and establishing a specific scientific community in sex and gender in transplantation, WIT’s impact will be felt for the coming decades. Continuing this high level of productivity has been made possible by financial contributions from our industry partners and with important administrative support from TTS.

## Data availability statement

The raw data supporting the conclusions of this article will be made available by the authors, without undue reservation.

## Author contributions

RM conceived and drafted this manuscript, which was edited by all authors. All authors discussed the contents and contributed to the final manuscript.

## Funding

The authors declare that this study received funding from Women in Transplantation Initiative of The Transplantation Society. The funder was not involved in the study design, collection, analysis, interpretation of data, the writing of this article, or the decision to submit it for publication.

## Acknowledgments

We thank the efforts of our administrator, Ms. Katie Tait, for her administrative support for the activities of *Women in Transplantation*.

## Conflict of interest

The authors are members of the WIT leadership and wish to thank their industry partners: Novartis, Sanofi, ThermoFisher-One Lambda, and Veloxis for their continued unrestricted support of activities in *Women in Transplantation*.

## Publisher’s note

All claims expressed in this article are solely those of the authors and do not necessarily represent those of their affiliated organizations, or those of the publisher, the editors and the reviewers. Any product that may be evaluated in this article, or claim that may be made by its manufacturer, is not guaranteed or endorsed by the publisher.
